# The Cluster of miR-143 and miR-145 Affects the Risk for Esophageal Squamous Cell Carcinoma through Co-Regulating Fascin Homolog 1

**DOI:** 10.1371/journal.pone.0033987

**Published:** 2012-03-23

**Authors:** Ran Liu, Juan Liao, Miao Yang, Jingyi Sheng, Hao Yang, Yi Wang, Enchun Pan, Wei Guo, Yuepu Pu, Sun Jung Kim, Lihong Yin

**Affiliations:** 1 Key Laboratory of Environmental Medicine Engineering, Ministry of Education, School of Public Health, Southeast University, Nanjing, China; 2 Huaian Center for Disease Control and Prevention, Huaian, China; 3 The First People's Hospital of Huaian, Huaian, China; 4 Department of Life Science, Dongguk University-Seoul, Seoul, Korea; National Cancer Institute, National Institutes of Health, United States of America

## Abstract

MicroRNAs (miRNAs), 18–24 nt non-coding RNAs, are thought to play important roles in cell proliferation, differentiation, apoptosis, and development. Recent studies suggest that some of the known microRNAs map to a single genomic locale within a single polycistronic transcript. But the roles of the cluster remain to be known. In order to understand the role and mechanism of a cluster of miR-143 and miR-145 in esophageal squamous cell carcinoma (ESCC), the association of mature miR-143 and miR-145 expression with the risk for esophageal cancer was evaluated in ESCC patients with a case-control study, and target protein regulated by mature miRNA was analyzed in ESCC cell lines with 3′UTR luciferase reporter assay. The expression levels of miR-143 and miR-145 were determined in 110 pairs of esophageal cancer tissues and adjacent normal tissues using real-time reverse transcription PCR. The relative expression of miR-143 and miR-145 were statistically different between cancer tissues and matched controls. The combined expression of miR-143 and miR-145 was significantly associated with the risk for esophageal cancer. Meanwhile, the reduced expression of two miRNAs in tumor patient was supposed to have a trend of lymph node metastases. The co-expression pattern of miR-143 and miR-145 was analyzed with Pearson correlation. It showed a significant correlation between these two miRNAs expression both in tissues and tumor cell lines. 3′UTR luciferase reporter assay indicated that Fascin Homolog 1 (FSCN1) could be co-regulated by miR-143 and miR-145. The protein level of FSCN1 showed no significant linear correlation with miR-143 and miR-145 expression in ESCC cell lines with Western blotting analysis. In conclusion, since miR-143 and miR-145 could regulate oncogenic FSCN1 and take part in the modulation of metastases, the result suggested the combination variable of miR-143 and miR-145 as a potential biomarker for earlier diagnosis and prognosis of esophageal cancer.

## Introduction

MicroRNAs (miRNAs), 18–24 nt non-coding RNAs, are thought to play important roles in cell proliferation, differentiation, apoptosis, and development in recent years [1], [2]. They are involved in endogenous post-transcriptional regulation function through perfect or imperfect complementary binding to specific sequences of target mRNAs, which they induce mRNA degradation or translational inhibition [3]. Many studies have demonstrated that the loss and gain of function of specific miRNAs may be key events in the disease process, particularly in the oncogenesis of cancer [4], [5], [6], [7].

**Figure 1:**
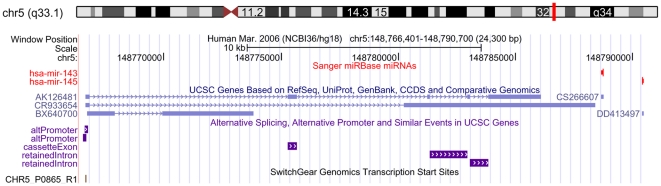
Schematic representations of miR-143 and miR-145 cluster in Chromosome. The human precursor mir143 and precursor miR-145 are located at the same intergenic region within about 2 kb on chromosome 5, which can be suggested to be a cluster. The mir143/miR-145 cluster might have a shared promoter with other genes from UCSC database.

Recent studies suggest that some of the known microRNAs map to a single genomic locale within a single polycistronic transcript [8], [9], [10]. The human mir143/miR-145 cluster contains 2 precursor miRNAs within about 2 kb on chromosome 5 (Figure 1). In this Figure, this cluster is located in the intergenic region and we predict that this cluster might have a shared promoter with other genes from UCSC database. The co-transcription of the two pre-miRNAs implicates that there are similar expression characteristics between miR-143 and miR-145. This cluster may play more important role in the cellular function through cooperative down-regulation of multiple targets compared with single miRNA function. Several studies explored that miR-145 or miR-143 played a tumor-suppressive role in various cancers [11], [12], [13], [14], [15], [16], [17], [18]. A large body of evidence detected by comparative genomic hybridization has established that 5q is a frequent loss segment in esophageal cancer with a loss frequency from 18% to 75% [19], [20], [21], [22], [23], [24], [25], [26]. Accordingly, the miR-143/miR-145 cluster located in 5q33 might be deleted or down-regulated in esophageal cancer. We hypothesize that the aberrant expression of mature miR-145 and miR-143 influence the regulation of target genes and involve in oncogenesis of esophageal cancer.

Moreover, FSCN1 was identified to be one of the targets of miR-145 [13]. Fascin, a 55 kDa actin-bundling protein encoded by FSCN1 gene, is an important regulatory element in the maintenance and stability of parallel bundles of filamentous actin and plays a central role in the regulation of cell adhesion, migration and invasion [27], [28]. Elevated evidences verified that fascin epithelial expression was significantly up-regulated in tumor tissues compared with adjacent benign tissues and the overexpression of fascin was associated with aggressive clinical course, poor prognosis and shorter survival of various tumors including prostate cancer, breast cancer, gastric cancer, renal cell carcinoma, pancreatic cancer, and etc. [29], [30], [31], [32], [33], [34], [35]. The overexpression of fascin in esophageal squamous cell carcinoma (ESCC) has been explored recently by several studies. These findings suggested that fascin was associated with the transformation and development of ESCC and implicated the potential of fascin as an early detection biomarker in ESCC [36], [37], [38], [39]. With predicted target genes result from TargetScan software, it is supposed that fascin can be regulated by miR-145 and miR-143 simultaneously. It implies that miR-143/miR-145 cluster may regulate the neoplasm process of ESCC through targeting fascin. In the present study, the association of mature miR-145 and miR-143 expression with esophageal cancer was determined in 110 pairs of esophageal cancer tissues and adjacent normal tissues, and target gene FSCN1 regulated by mature miRNA was analyzed in ESCC cell lines with Western blotting and 3′UTR luciferase reporter assay.

## Results

### Demographic characteristics

There are 110 patients with newly diagnosed, untreated esophageal squamous carcinoma recruited in the present study. The average age of the patients was 61.63±7.74 years. The ratio of male to female was 2.24. Of these patients diagnosed with pathological reports, 79 (71.8%) of 110 were diagnosed as well differentiated (I+II), 31 (28.2%) as poorly differentiated. Lymph node metastases were observed in 40 of 110 patients (36.4%).

### Differential expression of miR-143 and miR-145 in ESCC tissues and tissues adjacent to tumors

**Figure 2:**
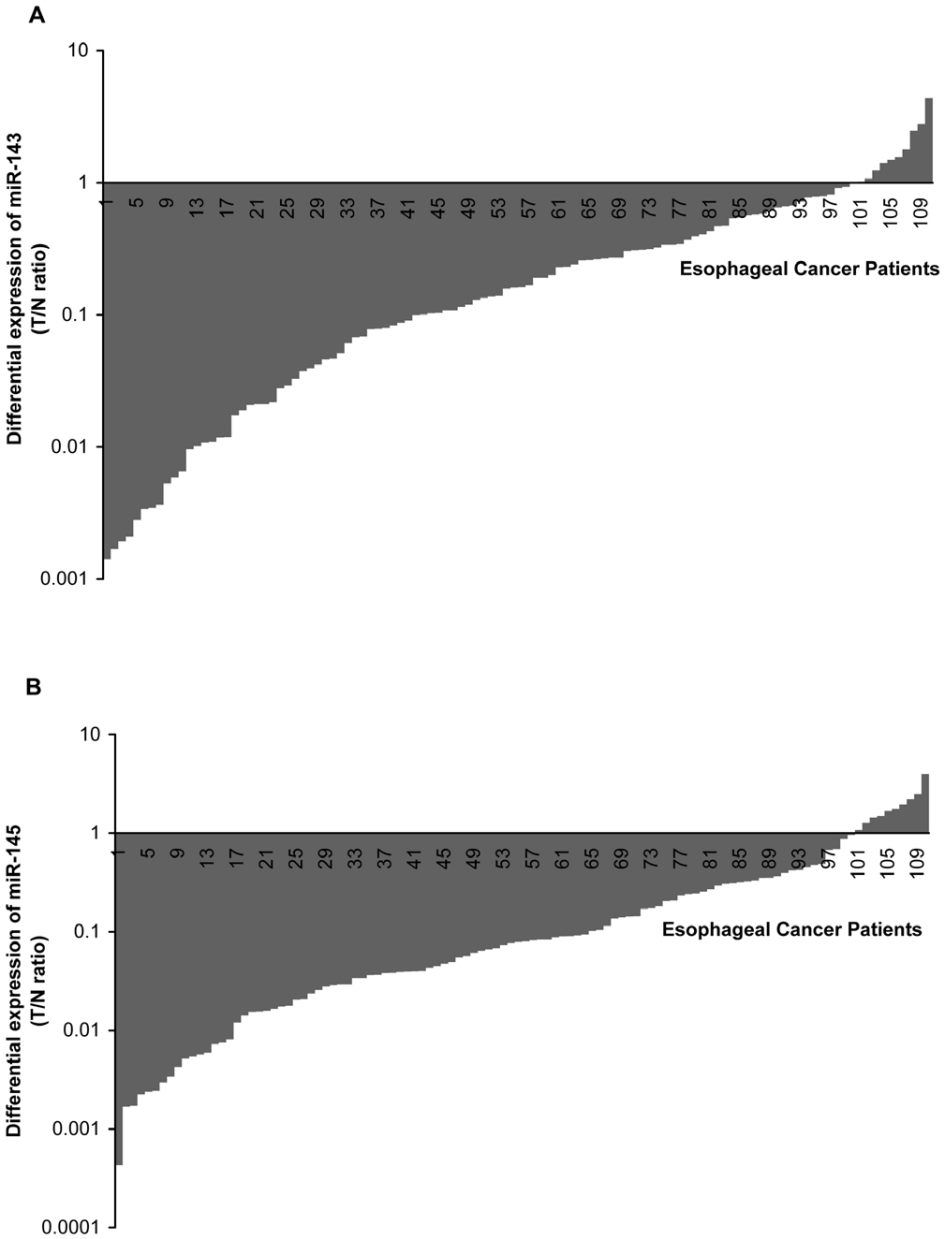
Differential expression of miR-143 and miR-145 in ESCC tissues and tissues adjacent to tumors. A represents the difference of miR-143 expression in each pair of tumor tissue (T) and adjacent normal tissue (N) from 110 ESCC patients. B represents the difference of miR-145 expression in each pair of tumor tissue (T) and adjacent normal tissue (N) from 110 ESCC patients.

Using the quantitative RT-PCR technique, the expression of miR-143 and miR-145 was determined in tumor tissues and tissues adjacent to tumors from cancer patients. Normalization to reference gene is essential for relative quantification of miRNA expression using real time PCR assay. U6 small nuclear RNA is highly conserved across species and the function of U6 snRNA has remained crucial to organism viability. Therefore, U6 snRNA shows relatively stable expression level in different tissues and cells [40]. Moreover, it doesn't participate in regulation network of microRNAs, which indicates that it is independent to microRNAs as an internal control. U6 snRNA has a comparable size with microRNAs, which guarantees the similar reverse-transcription efficiency between U6 snRNA and microRNAs. In sum, U6 fits the criterion of reference gene for real time RT-PCR and was used as an internal control in this study. With normalization of U6, the relative expression levels of miR-143 and miR-145 are presented in Figure 2. As relative expression data displayed the log-normal distribution, logarithmic transformation were performed in these data. As shown in Table 1, the relative expression of both two miRNAs showed statistical differences between cancer tissues and controls. Conditional logistic regression analysis revealed that significantly increased risk for esophageal cancer was associated with reduced expression of miR-143 and miR-145 (OR = 4.292, 3.367 respectively).

**Table 1:** Downregulation of miR-143 and miR-145 expression associated with a high risk of ESCC.

Group	Size	Relative expression level (95%CL)	Differential expression level	P value (t test)	OR (95%CL)
**miR-143**					
Tumor tissues	110	−1.943 (−2.439, −1.449)	−3.037 (−3.538, −2.536)	<0.0001	0.233 (0.124, 0.437)
Tissues adjacent to tumor	110	1.094 (0.615, 1.573)			
**miR-145**					
Tumor tissues	110	0.0587 (−0.364, 0.482)	−3.735 (−4.240, −3.229)	<0.0001	0.297 (0.176, 0.500)
Tissues adjacent to tumor	110	3.793 (3.367, 4.220)			

The associations of miR-143 and miR-145 with clinicopathological features were carried out with ANOVA or GLM analysis. There was no significant difference of miRNA expression according to age (p = 0.059, 0.240), gender (p = 0.663, 0.095), family history of cancer (P = 0.643, 0.653), tumor location (p = 0.514, 0.841), or differentiation (p = 0.099, 0.069). But the relative expression levels of miR-143 and miR-145 were different in the patients with Lymph node metastasis (p = 0.028, 0.017, Table 2 and Table 3).

**Table 2:** The associations of miR-143 expression with demographic and clinical characteristics.

Group	Size	miR-143 (Mean, 95%CL)	P value
**Age**			
<60	46	−3.601 (−4.413, −2.789)	0.059
≥60	64	−2.632 (−3.266, −1.999)	
**Gender**			
Male	76	−2.963 (−3.582, −2.344)	0.663
Female	34	−3.203 (−4.093, −2.313)	
**Family history of cancer**			
No	94	−3.086 (−3.641, −2.531)	0.643
Yes	16	−2.751 (−3.992, −1.510)	
**Tumor location**			
Upper part of esophagus	6	−3.587 (−6.486, −0.687)	0.514
Middle part of esophagus	93	−3.098 (−3.653, −2.543)	
Lower part of esophagus	11	−2.222 (−3.739, −0.704)	
**Differentiation**			
I and II	79	−2.776 (−3.344, −2.208)	0.099
III	31	−3.704 (−4.754, −2.654)	
**Lymph node metastasis**			
No	70	−2.618 (−3.215, −2.020)	0.028
Yes	40	−3.772 (−4.658, −2.885)	
**Smoking index**			
<400	47	−3.054 (−3.761, −2.347)	0.940
≥400	63	−3.015 (−3.736, −2.294)	
**Alcohol use**			
None or occasional	62	−2.458 (−3.247, −1.670)	0.043
Often	48	−3.486 (−4.129, −2.842)	

**Table 3:** The associations of miR-145 expression with demographic and clinical characteristics.

Group	Size	miR-145 (Mean, 95%CL)	P value
**Age**			
<60	46	−4.089 (−4.896, −3.283)	0.240
≥60	64	−3.480 (−4.138, −2.822)	
**Gender**			
Male	76	−3.450 (−4.054, −2.846)	0.095
Female	34	−4.372 (−5.305, −3.438)	
**Family history of cancer**			
No	94	−3.782 (−4.347, −3.218)	0.653
Yes	16	−3.455 (−4.628, −2.283)	
**Tumor location**			
Upper part of esophagus	6	−3.847 (−6.165, −1.529)	0.841
Middle part of esophagus	93	−3.781 (−4.318, −3.244)	
Lower part of esophagus	11	−3.283 (−5.682, −0.884)	
**Differentiation**			
I and II	79	−3.445 (−4.044, −2.846)	0.069
III	31	−4.474 (−5.417, −3.531)	
**Lymph node metastasis**			
No	70	−3.277 (−3.833, −2.721)	0.017
Yes	40	−4.536 (−5.512, −3.559)	
**Smoking index**			
<400	47	−3.189 (−3.831, −2.547)	0.013
≥400	63	−4.466 (−5.255, −3.677)	
**Alcohol use**			
None or occasional	62	−3.686 (−4.504, −2.867)	0.866
Often	48	−3.773 (−4.428, −3.118)	

T-test analysis was performed to determine the association between environmental factors and miRNA expression. As shown in Table 2 and Table 3, the result revealed that the expression level of miR-145 was significantly decreased in heavy smoking patients compared with non-smokers or occasional smokers (P = 0.013), while the expression level of miR-143 was significantly decreased in heavy drinking patients compared with non-drinkers or occasional drinkers (P = 0.043).

### Co-expression analysis of miR-143 and miR-145 cluster

**Figure 3:**
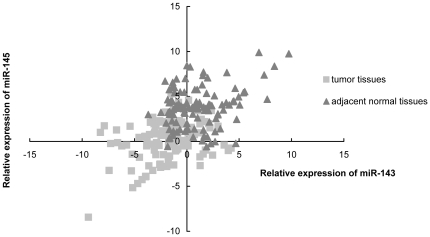
Scatter plot of miR-143 and miR-145 expression in ESCC patients. Relative expression of miR-143 and miR-145 normalized by U6 were evaluated with Pearson correlation analysis. Square represents samples from tumor tissues and triangle represents samples from normal tissues adjacent to tumor. The mixed sample set showed a significantly positive correlation between miR-143 and miR-145 expression.

The correlation of miR-143 and miR-145 was analyzed using Pearson correlation. With the log-transformed relative expression data from tumor tissues and tissues adjacent to tumor, Pearson correlation coefficient between miR-143 and miR-145 was determined as 0.322 (P = 0.0006) and 0.289 (P = 0.0022), respectively. When combined the expression datum of two groups into one, the analysis showed a significantly positive correlation between miR-143 and miR-145 as Figure 3 (R = 0.528, P<0.0001). It indicated that miR-143 and miR-145 were co-expressed in tumor tissues and tissues adjacent to tumor.

Logistic regression analysis was restarted according to the new independent variable defined as a combination value of miR-143 and miR-145. The linear regression equation of miR-143 and miR-145 was determined as follows. Y(miR-143) = 1.938X(miR-145)-4.157. And the combination value of miR-143 and miR-145 was defined as Y(combination) = 2.938X(miR-145). The result showed that significantly increased risk for esophageal cancer was associated with reduced expression of combination variable, which implied that the combination variable of miR-143 and miR-145 might be potential biomarker for earlier diagnosis of esophageal cancer.

### Expression of miR-143 and miR-145 cluster in ESCC cell lines

**Figure 4:**
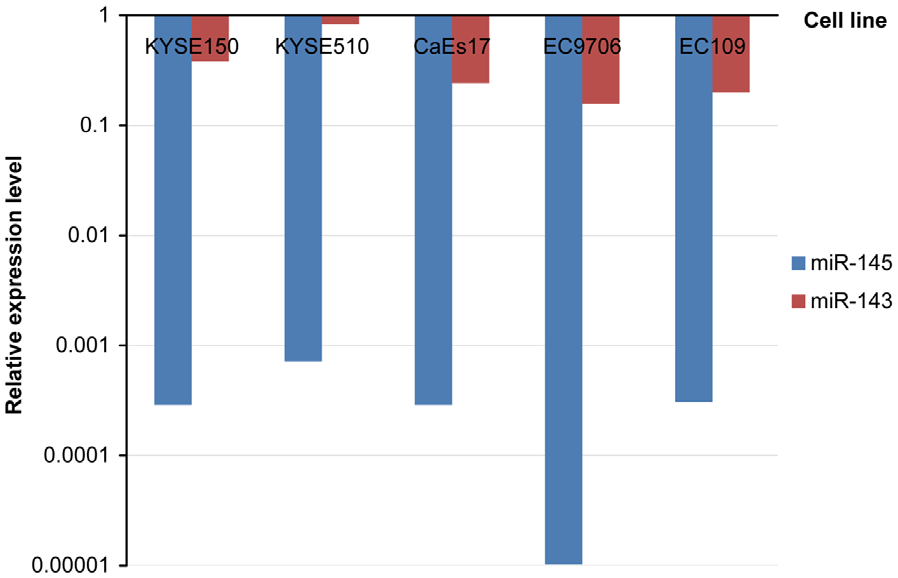
Relative expression of miR-143 and miR-145 in ESCC cell lines. Red bar and blue bar represent the relative expression of miR-143 and miR-145 normalized by U6 in five ESCC cell lines respectively. The correlation analysis suggested a co-expression pattern between miR-143 and miR-145.

The relative expression of miR-143 and miR-145 were determined in five ESCC cell lines normalized by U6 (Figure 4). EC9706 showed the lowest expression of miR-143 and miR-145, while KYSE510 had the highest expression. Since the expression of miR-145 was almost undetectable in EC9706, the correlation analysis between miR-143 and miR-145 was performed in ESCC cell lines except for EC9706. A significant positive correlation was found with 0.88 of R value. The result suggested a co-expression pattern between miR-143 and miR-145.

### Validation of FSCN1 as a target gene co-regulated by miR-143 and miR-145 using Luciferase Reporter Assay

**Figure 5:**
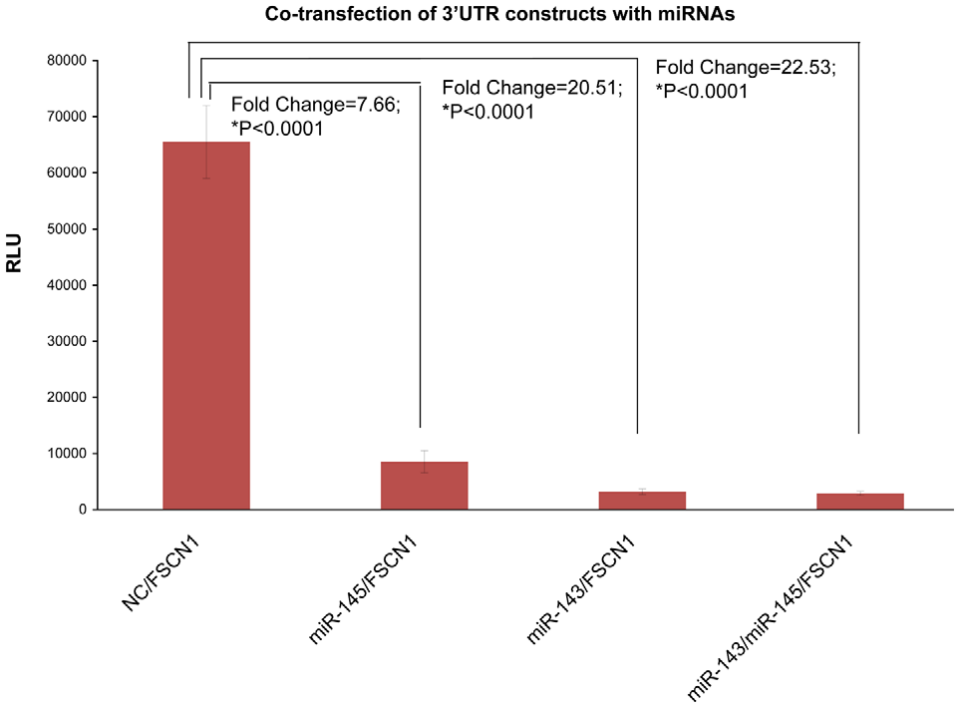
Luciferase reporter assay for 3′UTR of FSCN1 regulated by miR-143 and miR-145. Bar represents the RLU of negative control (NC), miR-145 mimic, miR-143 mimic, or miR-143 and miR-145 mimic mixture co-transfected with 3′UTR constructs of FSCN1. Asterisk (*) shows the statistically significant difference between negative control treatment and miRNA mimic treatment with ANOVA analysis based on factorial experiment design. Fold change shows the ratio of RLU from negative control treatment divided by RLU from miRNA mimic treatment.

Fascin homolog1 (FSCN1) was predicted to be regulated by miR-143 and miR-145 simultaneously with a miRNA target prediction tool (TargetScan Release 6.0). To confirm this predicted result, 3′UTR of FSCN1 was cloned to construct luciferase reporter vector. Figure 5 showed the luciferase signal results from co-transfection of 3′UTR constructs from FSCN1 with miR-143 mimic, miR-145 mimic, miR-143 and miR-145 mimic mixture or negative control. ANOVA analysis based on factorial experiment design indicated that both miR-143 and miR-145 inhibited 3′UTR expression of FSCN1 significantly (P<0.0001). The miR-143 showed 2-fold higher inhibition efficiency than miR-145. Co-transfection of miR-143 and miR-145 showed a synergistic effect on suppressing the expression of FSCN1 (P<0.0001).

### Association of FSCN1 with miR-143/miR-145 cluster using Western Blotting assay

**Figure 6:**
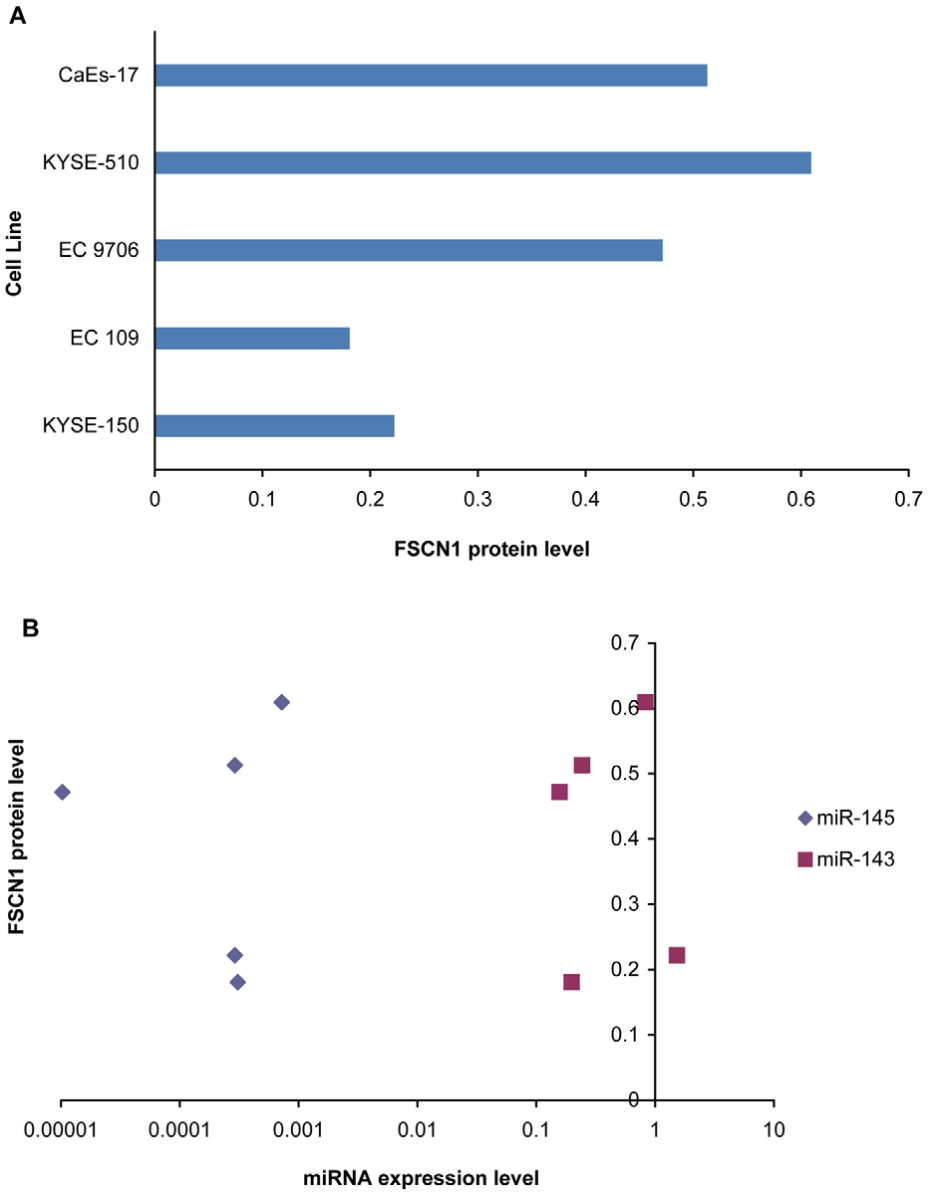
Association of FSCN1 with miR-143/miR-145 cluster. A represents the protein level of FSCN1 normalized by β-actin in five ESCC cell lines with different miR-143 and miR-145 expression level using Western Blotting assay. B represents the scatter plots of miR-143 (square) and miR-145 (diamond) expression correlated with FSCN1 protein in ESCC cell lines.

Since Fascin homolog1 (FSCN1) was confirmed to be regulated by miR-145 and miR-143 simultaneously with 3′UTR luciferase reporter assay, the protein levels of FSCN1 in five ESCC cell lines were determined by Western Blotting to explore the actual association between FSCN1 protein and miR-143/miR-145 cluster. As shown in Figure 6, normalized by β-actin, FSCN1 had a highest level in KYSE-510 and a lowest level in EC109. Correlation analysis of these two miRNAs expression and FSCN1 showed no statistical significance.

## Discussion

The role of miRNA in tumorigenesis has been extensively studied in recent years. A early evidence showed that miRNAs were commonly located in fragile sites on chromosomes, preferential sites of translation, deletion and amplification that are often altered in cancers [41]. 5q, located with miR-143/miR-145 cluster, was found to be frequently lost in esophageal cancer detected by comparative genomic hybridization [23]. Hu et al identified 5q with very high frequency (>/ = 75%) loss of heterozygosity from a genomewide scan of esophageal squamous-cell carcinoma in a high-risk Chinese population [19]. Montesano et al and Moskaluk et al all explored 5q was frequent target of deletion in esophageal cancer and may harbor novel tumor suppressor genes [21], [22]. The cluster of miR-143 and miR-145 is predicted within about 2 kb on chromosome 5 plus chain by miRGen clusters tool (http://www.diana.pcbi.upenn.edu/cgi-bin/miRGen/v3/Cluster.cgi). And two alternative promoters are found to be located within 24 kb upstream of miR143–miR145 cluster, which suggests that this cluster may be co-transcribed and play more important role in biological function than other single miRNAs. Several researches showed that the expression levels of miR-143 and miR-145 were downregulated in multiple tumor tissues including lung cancer, gastric cancer, breast cancer, colorectal cancer and etc. when compared with adjacent normal tissues [11], [42], [43], [44]. However, whether miR-143 and miR-145 are co-transcribed and what function this cluster has remains to be understood.

In the present study, we found that the relative expression of both two miRNAs showed statistical differences between cancer tissues and controls. Moreover, they showed a significant correlation between these two miRNAs expression both in tumor tissues and tumor cell lines. It suggested that miR-143 and miR-145 were co-expressed descendingly in esophageal cancer. The combined expression of miR-143 and miR-145 was significantly associated with the risk for esophageal cancer. Meanwhile, the reduced expression of two miRNAs in tumor patient was supposed to have a trend of lymph node metastases.

In order to understand the process of miR-143 and miR-145 cluster affecting the development and metastases of esophageal cancer, we searched for the target gene regulated by miR-143 and miR-145 simultaneously. FSCN1 was predicted to be co-targeted by miR-143 and miR-145 with TargetScan and the co-regulation relationship of miR143 and miR-145 then was confirmed using 3′UTR luciferase reporter assay. Fascin can organize filamentous actin into bundles and plays a role in the formation of microspikes, membrane ruffles, and stress fibers. It induces the formation of membrane protrusions, and promotes cell motility and migration (http://www.uniprot.org/uniprot/Q16658#section_comments). The overexpression of fascin in esophageal squamous cell carcinoma (ESCC) has been described [36]. Using RNA interference (RNAi), fascin was stably silenced, which resulted in a suppression of cell invasiveness [38]. FSCN1 was recently identified to be a direct target gene regulated by miR-145 with luciferase reporter assay [13]. And our result also indicated that 3′UTR of FSCN1 could be co-regulated by miR-143 and miR-145. With these researches, we suggested that miR-143 and miR-145 could control oncogenic FSCN1 and take part in the modulation of metastases. Even though our present result showed no significant correlation between miR-143 and miR-145 co-expression and protein level of FSCN1, it was supposed that FSCN1 could be regulated by multiple miRNAs and may not reveal linear correlation with miR-143 and miR-145 co-expression.

In conclusion, miR-143 and miR-145 were suggested to be co-expressed descendingly in esophageal cancer in the present study. The combined expression of two miRNAs in tumor patient showed a negative association with lymph node metastases. The cluster of miR-143 and miR-145 should take part in the modulation of metastases through targeting oncogenic FSCN1. The result suggested the combination variable of miR-143 and miR-145 as a potential biomarker for earlier diagnosis and prognosis of esophageal cancer.

## Materials and Methods

### Study Subjects and Ethics Statement

110 patients with newly diagnosed, untreated esophageal cancer were recruited in the present study. All cases were from Huaian county of Jiangsu province, China. Patients were newly diagnosed with histologically confirmed primary cancer and previously untreated (no radiotherapy or chemotherapy) ESCC from October 2008 to December 2010. During the period of recruitment, each subject was scheduled for an interview after informed consent was written, and a structured questionnaire was administered by the interviewer to collect information about demographic data and risk factors such as smoking status, alcohol use etc.. Esophageal cancer tissues and tissues adjacent to the tumors were macro-dissected from each subject during operation. In order to ensure a high proportion of tumor cells when collecting tumor tissue, the site and range of tumor were determined and 0.5 m^2^ of tumor tissue outward from the center was captured only with the objects of approximately 1 centimeter in diameter and larger. For normal epithelial cells collection, 0.5 m^2^ of esophagus was dissected further than 5 centimeters from the tumor edge and then muscle layer and connective tissue were removed thoroughly to get the high purity of esophageal epithelia. Within half an hour after tissues dissected, the samples were stored into RNAlocker reagent for miR-143 and miR-145 expression analysis using real time RT-PCR assay. The population study was approved by the institutional review board named as “IRB of Southeast University Affiliated Zhongda Hospital” in Nanjing, China. IRB of Southeast University Affiliated Zhongda Hospital approved the design of esophageal cancer study including tissue samples collection.

### Cell lines and culture

All human ESCC cell lines used in the present study were established cell lines as follows. EC9706 and EC109 were purchased from Shanghai Tiancheng Technology Co., Ltd. CaEs-17 were purchased from Nanjing KeyGen Biotech Co., Ltd.. KYSE150 and KYSE510 were purchased from Cell Center of Shanghai Institute of Life Science, Chinese Academy of Science. Cell lines were cultured in RPMI1640 medium (Invitrogen) supplemented with 10% heat-inactivated fetal bovine serum (Invitrogen) and 1% penicillin/streptomycin (Sigma-Aldrich). Cells were incubated in a humidified incubator at 37°C and 5% CO2. The trypsinized cells were harvested for miRNAs expression and fascin analysis using real time RT-PCR and Western blotting respectively.

### Isolation of Total RNA and Total Proteins

Esophageal tissues were homogenized in 1 ml of TRIZOL reagent per 30–50 mg of tissue. Cell pellets were homogenized in 1 ml of TRIZOL reagent per 10^6^ cells. Total RNA was isolated from homogenate according to the manufacturer's instructions, extracted with chloroform, precipitated with isopropyl alcohol, washed with ethanol, and dissolved in RNase free water. The concentrations of RNA were determined spectrophotometrically by monitoring UV absorbance at 260 nm. Purity was assessed by the absorbance ratio 260/280 nm.

Total cell lysates were prepared in RIPA buffer [50 mmol/L TrisHCl, pH 8.0, 150 mmol/L NaCl, 1% (vol/vol) Nonidet P-40, 0.5% (wt/vol) sodium desoxycholate, 0.1% (wt/vol) SDS] containing the complete protease inhibitor cocktail. Protein concentration was determined using the Bio-Rad protein assay (Bio-Rad) with BSA as standards.

### Quantitative Reverse Transcription PCR

Quantitative reverse transcription PCR analysis of miRNA expression was carried out using a 7300 Real Time PCR System (Applied Biosystems). U6 small nuclear RNA was used as an internal control to normalize RNA input. Briefly, 10 µl reverse transcription (RT) reaction mixture contained 0.5 µg of total RNA, 5 nM of miRNA-specific stem-loop RT primer, 500 µM of dNTP mixture, 2 µl of 5×RT buffer, 10 U of Ribonuclease Inhibitor (Sigma), 100 U of MMLV (Promega), and RNase-free water to final volume. The RT reaction was performed as follows: 16°C for 30 minutes, followed by 42°C for 60 minutes, heated to 85°C for 5 minutes and then stored at −20°C. Specific reverse transcription primers for miR-143 and miR-145 cDNA synthesis were 5′- GTC GTA TCC AGT GCG TGT CGT GGA GTC GGC AAT TGC ACT GGA TAC GAC AGG GAT -3′ and 5′- GTC GTA TCC AGT GCG TGT CGT GGA GTC GGC AAT TGC ACT GGA TAC GAC GAG CT- 3′ respectively. Specific reverse transcription primer for U6 was 5′- GTC GTA TCC AGT GCA GGG TCC GAG GTG CAC TGG ATA CGA CAA AAT ATG GAA C -3′.

The real-time PCR was carried out in 96-well plates (Axygen) with a final volume of 20 µl using SYBR Green I dye. The PCR reaction components were 1 µl of cDNA synthesized as above, 10 µl of 2×SYBR Green PCR Master Mix, and 0.6 µM of each pair of oligonucleotide primers. Thermal cycling conditions were 95°C for 5 minutes, followed by 40 cycles of 95°C for 15 s, 60°C for 60 s. A dissociation curve analysis was added after the final PCR cycle to evaluate the presence of nonspecific PCR products and primer dimers. Specific primers for miR-145 cDNA amplification were 5′- TCG GTC CAG TTT TCC CAG -3′ (sense) and 5′- AGT GCG TGT CGT GGA GTC -3′ (antisense) with the product size of 65 bp. Specific primers for miR-143 cDNA amplification were 5′- AGC GTG TGT CGT GGA GTC -3′ (sense) and 5′- TCG TGA GAT GAA GCA CTG TAG -3′ (antisense) with the product size of 63 bp. Primer sequences for U6 were 5′- TGC GGG TGC TCG CTT CGG CAG C -3′ (sense) and 5′- CCA GTG CAG GGT CCG AGG T -3′ (antisense) with the product size of 144 bp. The amount of template cDNA was expressed by a threshold cycle (Ct) that was determined by the amplification curve (exponential phase). The parameter Ct was defined as the fractional cycle number at which the fluorescence caused by the binding of SYBR Green dye to double-stranded (ds) DNA reaches detection threshold. The miRNA levels were compared between subjects by a comparative Ct method with separate tubes, as described elsewhere [45]. Briefly, the individual level of initial target cDNA was expressed as the difference in Ct between the target and an internal control U6 (ΔCt).

### Luciferase reporter assay

TargetScan release 6.0 was used to predict co-regulated target genes of miR-143 and miR-145. For confirmation of direct target binding, the 3′UTR of FSCN1 identified by TargetScan was cloned into a 3′UTR_vector (GeneCopoeia Genomics). EC9706 cells were seed into a 96-well plate with a concentration of 10 thousand cells each well in 100 µl total volume and incubated for 24 hours. Cells were then co-transfected with miR-143 mimic, miR-145 mimic, miR-143 and miR-145 mimic mixture or negative control and 3′UTR_vector. 100 ng of 3′UTR_vector was mixed with 1.5 pmol of miRNA mimic or negative control in 10 µl of OPTI-MEM. 0.25 µl of lipofectamine2000 was diluted into 10 µl of OPTI-MEM and added into the former mixture after incubated for 5 minutes. When incubated for another 20 minutes, 20 µl of transfection mixture and 80 µl of antibiotics-free RPMI1640 media were added into the 96-well plate and incubated at 37°C and 5% CO2 for 24 hours. The substrate solution was used for luciferase assay. After incubation of 30 minutes, the signal from each well was read with Mithras LB 940 (Berthold Technologies). Luciferase signal ratio for specific miRNA over negative control was calculated for each construct.

### Western Blotting of FSCN1

The protein levels of FSCN1 were determined in the ESCC cell lines by western blotting. Twenty µg of the isolated total proteins were mixed with 2× loading buffer and boiled for 5 minutes. SDS-PAGE was performed with 12% separation gel concentration and 5% spacer gel concentration. β-actin (45 KD) and FSCN1 (55 KD) were transferred PVDF membrane at 250 mA for 1.5 hour. Blocking was performed with 5% nonfat milk for 2 hour at room temperature. Primary mouse anti-human β-actin IgG and rabbit anti-human FSCN1 IgG titers were optimized at 1∶5000 and 1∶1000 respectively, and incubated with the membrane for 1.5 hour at room temperature. After washing with PBS buffer, the membrane was incubated with the secondary antibody (dilution 1∶5000) for 1.5 hour at room temperature. Identical substrates were used in each assay for enhanced chemiluminescence detection (Pierce) according to the manufacturer's protocol.

### Statistical Analysis

The prediction of microRNA targets was carried out through TargetScan (release 6.0, http://www.targetscan.org/) by searching for the presence of conserved and poorly conserved 8 mer and 7 mer sites that match the seed region of microRNA [46].

Analysis of variance (ANOVA), Chi-square test, and logistic regression analysis were used to determine the statistical differences between tumor tissues and tissues adjacent to the tumors at a significance level of 5%. The correlation coefficients of miR-143 and miR-145 were calculated using the Pearson correlation. The relationships between miRNAs expression and FSCN1 expression were analyzed using the Pearson correlation. All statistical analysis mentioned above were performed using SAS software (Version 9.0).
